# Communicating clearly about data sharing in genomics

**DOI:** 10.1186/s40246-025-00784-z

**Published:** 2025-07-14

**Authors:** Donrich Thaldar, Diya Uberoi, Adrian Thorogood, Richard Milne, Ainsley J. Newson, Alison Hall, David Glazer, Paul Esselaar, Yann Joly

**Affiliations:** 1 School of Law, University of KwaZulu-Natal, Durban, South Africa; 2 Centre of Genomics and Policy, Institute of Genomics Medicine, McGill University, Montreal, QC Canada; 3 Terry Fox Research Institute, Vancouver, BC Canada; 4 Wellcome Connecting Science, Wellcome Sanger Institute, Hinxton, UK; 5 Kavli Centre for Ethics, Science, and the Public, University of Cambridge, Cambridge, UK; 6 Sydney Health Ethics, Sydney School of Public Health, Faculty of Medicine and Health, University of Sydney, Sydney, Australia; 7 PHG Foundation, University of Cambridge, Cambridge, UK; 8 Verily Life Sciences, South San Francisco, CA USA

**Keywords:** Genomic data sharing, Data visiting, Cross-border data transfer

## Abstract

In the field of genomics, the secure and responsible sharing of data across institutions and borders is critical for advancing research and improving healthcare. However, challenges such as inconsistent terminology, data localization requirements, and cross-border data transfer regulations impede collaboration and innovation. To address these barriers, the Global Alliance for Genomics and Health (GA4GH), a global standards-setting organization in genomics, has developed a standardized lexicon of key terms for data sharing, including the nascent terms *data visiting* and *federated data analysis*. These definitions aim to improve communication within the genomics community by ensuring a consistent understanding of complex processes, addressing challenges like data localization and cross-border transfer. This article introduces these recently developed data sharing-related terms and considers their implications for data governance and global health research.

**Supplementary Information:**

The online version contains supplementary material available at 10.1186/s40246-025-00784-z.

## Introduction

In the rapidly evolving field of genomics, data sharing across institutions and borders is widely considered essential to advancing research and improving healthcare outcomes [1]. Yet, legal, technical, and ethical barriers still impede the secure sharing of genomic and health data globally [2]. Formed in 2013, the Global Alliance for Genomics and Health (GA4GH) unites over 500 leading organizations from healthcare, research, ethics, government, and technology to address these challenges [3]. GA4GH develops technical standards, policy frameworks, and open-source tools that promote the voluntary and secure use of genomic and related health data [4]. 

By building consensus among all actors in the genomics field, GA4GH aims to create a common understanding of terms which will assist in sharing genomic data globally in a secure manner, with respect for data privacy and health research ethics and law. The creation of this environment is an essential precursor to enabling research that can advance health for all. GA4GH envisions a future where evidence-based applications of genomics are a routine part of healthcare, enabling the development of personalized medicine to improve patient outcomes. However, barriers remain—data are often siloed, stored in incompatible systems, and governed by complex legal and ethical rules [5]. 

A key challenge in this field is the inconsistent use of terminology. Terms such as *data visiting* and *federated data analysis* are frequently used in genomics literature, but often with varying meanings [6–10]. This lack of clarity can lead to confusion and hinder collaboration across institutions. Therefore, clarifying and standardizing these terms in an openly accessible GA4GH lexicon is crucial. Doing so can align global efforts, improve communication, and help researchers, institutions, and policymakers work with a common understanding.

This article introduces these nascent data sharing-related terms and considers their implications for data governance and global health research.

## Methodology

The Data Visiting Study Group (the Study Group) was formed under the GA4GH’s Regulatory and Ethics Workstream (REWS) in September 2023. The group brings together over 50 multidisciplinary scholars from over 25 jurisdictions worldwide. Over 12 months, the group convened a series of regular meetings and workshops, complemented by multiple online reviews, to develop the lexicon presented in this article. Work on the lexicon comprised four forms: specific Study Group meetings, presentations of the study group’s work at REWS meetings, a presentation and discussion of the study group’s work (including draft definitions) at a GA4GH conference, and asynchronous work within a shared document (Google Docs). Four meetings were held in total, with multiple between-meeting interactions. As is standard for GA4GH, all documentation was available to all interested members. Study Group members could actively and directly edit terms.

To help develop a baseline lexicon for comment, the group undertook an informal initial scan of the literature. This scan allowed the group to identify a set of terms relevant to genomic data sharing for which there was no shared or consistent meaning. However, this was not a literature review and the literature identified was not formally analyzed. The Study Group also felt that, in general, any previously published definitions, which were neither consensus definitions nor endorsed by any professional body, were unclear or did not comply with ethical or regulatory norms. As a result, the group determined that no existing definition could be endorsed and that new definitions were required.

In developing new definitions, the Study Group was guided by three criteria:

1. the use of plain language;
2. alignment with how terms are understood and applied in practice (in the view of the expert group members); and
3. for definitions to be ‘flat’ and factual, avoiding the inclusion of value-based assumptions or perceived best practices.

The lexicon was developed iteratively and through consensus. Broad concepts and initial draft terms were presented in April 2024, a first draft of the full lexicon was presented in May 2024, and a revised version in June 2024. The lexicon was finalized by the Study Group in July 2024 and then formally approved by REWS at its general meeting in September 2024. Formal REWS approval is required to ensure consistency across GA4GH’s standard-setting process. As noted, in addition to discussion at meetings and workshops, the study group carried out its work collaboratively by editing and commenting on a shared online document.

The final definitions presented below represent the outcome of expert consensus within the Study Group and are endorsed by the GA4GH’s Regulatory and Ethics Workstream. A copy of the lexicon is provided in Appendix 1.

## The newly defined terms

### Data sharing

#### Definition

*Data sharing is where one party*,* the provider*,* provides access to data to another party*,* the user*,* with the purpose of the user using the data*,* as agreed with the provider.*

In data sharing, *shared data* can be used for a variety of tasks, including cleaning the data to remove inconsistencies, reformatting it to integrate with other datasets, combining it with additional data to generate new insights, annotating it with metadata for clarity, training models, or performing analyses that generate actionable results [11]. These processes are fundamental to deriving insights from genomic data, contributing to precision medicine and innovative research in healthcare.

Typically, the provider and user are institutional actors—such as universities, research consortia, or companies—rather than individual researchers. As such, data sharing most often occurs between two independent entities and is commonly governed by a written data sharing agreement. While such an agreement is widely recognized as a key legal instrument that should be carefully drafted [12–14], it is considered best practice rather than a constitutive element of data sharing itself. For example, a provider and user can agree to share data through an exchange of emails or even verbally. As long as there is an intention to be legally bound, such an agreement would constitute a binding legal contract, even though it would lack the comprehensiveness and detailed provisions that a professionally drafted formal agreement would provide. For this reason, the requirement of a formal written agreement was not included in the GA4GH definition.

The GA4GH definition is closely aligned with other definitions, such as the definition provided in the NIH Grants Policy Statement, which describes data sharing as “the act of making scientific data available for use by others (e.g., the larger research community, institutions, the broader public), for example, via an established repository.” [15] Both definitions focus on enabling the use of data by others and refrain from requiring a formalized agreement as part of the definitional core. However, the NIH definition conceptualizes data sharing as a unidirectional act—“making data available”—without acknowledging the inherently relational and mutual character of the activity. In contrast, the GA4GH definition explicitly includes an agreement between the provider and the user, thereby underscoring the collaborative nature of data sharing and enhancing clarity in contexts where control, responsibility, and permitted uses are of legal and ethical importance.

The GA4GH definition is also conceptually aligned with the definition of ‘data sharing’ adopted by the New South Wales (Australia) Infrastructure Data Management Framework (IDMF) and the Australian Capital Territory’s Government Data Sharing Policy, the latter of which defines ‘sharing’ as ‘making information or data available to another agency, organisation or person under agreed conditions.’ [16] While substantially similar, the GA4GH definition improves on this formulation in terms of precision and economy of language.

A further positive attribute of the GA4GH definition lies in the introduction of role-specific terminology: *provider* and *user*. These terms are used consistently across the full set of definitions developed by the GA4GH Data Visiting Study Group, thereby fostering coherence and ease of cross-referencing within a broader data governance framework.

In summary, the GA4GH definition of data sharing offers several important positive attributes. It:

- highlights the consensual nature of data sharing through explicit reference to agreement between parties;
- introduces neutral, role-specific terminology (*provider*, *user*) used consistently across related definitions;
- avoids the inclusion of policy-specific or normative elements that may not be universally relevant; and
- adheres to principles of clarity, neutrality, and practical relevance in the articulation of core terms.

### Data visiting

#### Definition

*Data visiting is a form of data sharing in which shared data is analyzed within the provider’s computing environment*,* whether through human or computational agents.*

In data visiting, rather than data being downloaded and analyzed in the user’s own environment, the data remains under the provider’s control throughout the analytical process. Data visiting supports a wide range of analytical activities, including querying, visualizing, manipulating, extracting features, and/or running simulations. Importantly, “the provider’s computing environment” does not require the data provider to be the legal owner of the physical infrastructure; it simply refers to any computing environment effectively under their control. This environment could be outsourced or hosted by a third-party provider, so long as the data provider maintains control over access and data handling within it. It is also important to note that the data provider’s computing environment can either be local or cloud-based. In other words, data visiting is not dependent on the physical location of the shared data—it can be stored on the cloud or on local servers. Rather, it is the action of the user analyzing the data in the provider’s computing environment—irrespective of where it is—which constitutes data visiting.

While data visiting is increasingly relevant to modern data sharing practices, our examination of existing literature and institutional sources revealed limited formal recognition of the term. Indeed, only one other entity had considered a definition for data visiting: the LINEAGE consortium, which adopted Weise et al.’s introductory description, characterizing data visiting as “a form of data access whereby data users view and analyze the data within the computing environment(s) of the data provider(s)” [10]. Beyond this, references to data visiting in the scholarly literature were limited and conceptually inconsistent [6–9], confirming that data visiting remains, for academic purposes, largely a neologism.

The GA4GH definition differs from Weise et al.’s formulation in one key respect: the definition includes *viewing* the data as part of the definition of data visiting, whereas the GA4GH definition requires only that *analysis* occur within the provider’s computing environment. This divergence reflects an important empirical observation. In current practice, many organizations that implement data visiting architectures permit users—whether human researchers or automated agents—to perform analyses without necessarily granting them the ability to view the underlying data directly. The capacity to view data may be restricted for legal, ethical, or security reasons, and is therefore not a reliable or universal feature of data visiting models.

Empirical practice demonstrates that the extent to which data visitors can *view* shared data varies significantly across implementations. In models such as those of the UK Biobank [17] and the All of Us Research Program [18]approved researchers are permitted to view individual-level data—including raw genomic and phenotypic values—within secure environments, although identifiers are pseudonymized and access to sensitive fields is tightly controlled. By contrast, models like Australian Genomics [19] and ELIXIR [20] allow only limited viewing: researchers may interact with pseudonymized data or aggregated outputs, but access to raw individual-level data is either prohibited or subject to exceptional governance approval. At the other end of the spectrum, Apheris [21] exemplifies a privacy-maximizing approach in which data visitors cannot view any part of the underlying dataset; they submit computational queries or algorithms and only receive processed outputs, such as model parameters or summary statistics. These examples confirm that while analysis in the provider’s computing environment is a common and defining feature, the ability to view data is not consistent across models. This diversity supports the GA4GH Study Group’s decision to define data visiting in terms of *analysis*, rather than *viewing*, thereby aligning the definition with current practice while maintaining flexibility across governance models.

### Federated data analysis

#### Definition

*Federated data analysis refers to data visiting involving multiple providers.*

When analysis of results depends on reasoning across data from multiple sources (potentially including data directly controlled by the user), and those sources don’t allow moving data to a central location, federated data analysis is required. This form of collaboration allows researchers to share insights, results, and computations without transferring the shared data, making it ideal for sensitive health research [8, 17]. Federated data analysis includes *federated learning*, which is a machine learning approach where a global model is trained across multiple datasets that remain distributed across different locations [22].

The GA4GH definition of federated data analysis is both concise and consistent with terminology established elsewhere in the definitions set, particularly in the use of *providers*—a term also employed in the definition of data sharing. This internal consistency aids clarity and avoids conceptual ambiguity. Although not formally defined, the ELIXIR Cloud and AAI GA4GH Driver Project uses the term in a manner that is clearly aligned with the GA4GH definition, describing federated data analysis as research conducted across datasets without the data leaving its host, which captures the essence of multiple providers retaining control [20]. The NIH, in Funding Opportunity RFA-HG-17-011, defines federated data analysis as participation in a “federated genomic data commons ecosystem” [23]. While substantively similar, the NIH formulation introduces the concept of a *data commons ecosystem*, which the GA4GH Data Visiting Study Group considered to be potentially broader and less precise. By contrast, the GA4GH definition deliberately retains the terminology of *providers*, ensuring continuity across definitions and enhancing conceptual clarity.

### Remote data interrogation

#### Definition

*Remote data interrogation is a restricted type of data visiting where the user can only query*,* but not view*,* the shared data within the provider’s computing environment and can only view the results of the query.*

This method provides an additional layer of security over data visiting by imposing additional researcher restrictions, ensuring that sensitive or restricted datasets are not directly exposed to the user [24]. Although the term is not yet well established in the literature, a small but growing body of work reflects the underlying concept, typically in the context of query-based access models that limit user interaction to predefined outputs without granting visibility of the raw data. Such models are increasingly employed in federated and privacy-preserving infrastructures to facilitate data analysis while reducing disclosure risk [25–27]. Importantly, by defining remote data interrogation as a specific type of data visiting in which *viewing* is excluded by definition, the GA4GH framework offers a more precise typology than other previous formulations, which treat viewing and analysis as separate elements of data visiting [10]. In contrast, our approach recognizes that in current practice, analysis often occurs without data visibility, and accordingly distinguishes remote data interrogation as a distinct, operationally relevant category within the broader data visiting paradigm.

### Cross-border data transfer

#### Definition

*Cross-border data transfer is the transfer of data across jurisdictions.*

This is a critical concern in genomic research, where researchers may need to access data stored in different countries, states, and territories. While most legal frameworks impose conditions for lawful cross-border transfers—typically requiring adequate protection in the recipient jurisdiction or the data subject’s consent—they do not specify what *transfer* entails in technical terms. A review of data protection legislation in jurisdictions such as Brazil [28], the EU [29], India [30], Israel [31], Kenya [32], Mexico [33], New Zealand [34], Nigeria [35], South Africa [36], South Korea [37], Turkey [38], and the UK [39] reveals that while the term *transfer* is used in the jurisdiction’s data protection legislation, it is left undefined. As a result, key questions—such as whether temporary viewing or short-term processing constitutes a transfer—remain unanswered in these jurisdictions. Exceptions do exist: for example, the European Data Protection Board (EDPB) has issued guidance defining *transfer* broadly as “disclosing by transmission” or otherwise “make… available” to another party in a third country [40]. However, this approach is not universally followed, and in most cases, regulatory interpretation has yet to be developed. The GA4GH definition therefore adopts a cautious and jurisdiction-neutral approach, clarifying only that *cross-border* refers to data crossing jurisdictional boundaries, while deliberately leaving *transfer* undefined. This reflects the understanding that the legal meaning of *transfer* is a technically complex and evolving issue, one more appropriately addressed through future comparative legal research and jurisdiction-specific regulatory guidance.

### Data localization

#### Definition

*Data localization refers to legal or ethical rules*,* or efforts to comply with such rules*,* that aim to keep data within a jurisdiction that governs such data.*

To protect their citizens’ personal information, many countries have enacted legal and ethical requirements to ensure that sensitive health or personal data remains within national borders, preventing its transfer to other jurisdictions [41, 42]. These requirements negatively impact data sharing, particularly in international collaborations, as they limit researchers’ flexibility and increase the complexity of global research, especially in multi-jurisdictional projects. Institutions that want to share data may use data visiting in an attempt to comply with localization requirements.

## General discussion

The introduction of GA4GH’s new terminology, including *data visiting* and *remote data interrogation*, presents important opportunities for standardizing communication in the genomics field. By providing concise, neutral, and interoperable definitions, the lexicon enables researchers, institutions, funders, and regulators to speak the same conceptual language—regardless of jurisdictional or technical context. This shared terminology provides a foundation for international collaboration, supports regulatory clarity, and lays the groundwork for future technical and policy standardization.

A key strength of the lexicon is its conceptual coherence. All definitions are anchored in the central relationship between a *provider* and a *user*, allowing related terms such as *data sharing*, *data visiting*, *remote data interrogation*, and *federated data analysis* to interlock without ambiguity. The decision to retain consistent terminology across definitions—particularly the use of *provider* and *user*—avoids the confusion introduced by less specific formulations such as “data commons ecosystem” and reinforces the operational clarity of the framework. Rather than encoding technical standards or best practices, the definitions are deliberately minimalistic, offering just enough conceptual specificity to enable dialogue while remaining agnostic as to implementation models. This neutrality makes the lexicon adaptable across a wide variety of legal systems, infrastructures, and governance frameworks.

The definitions also support modular thinking about data governance, enabling researchers and policymakers to distinguish between related but distinct practices. For example, treating *remote data interrogation* as a specific type of *data visiting*—one in which viewing is excluded—allows for clearer technical and legal design, especially in environments where privacy concerns or data localization laws demand heightened restrictions. Similarly, recognizing *federated data analysis* as involving multiple providers builds on the core data visiting concept without introducing inconsistencies. This layered approach supports scalable innovation, from isolated query models to complex, cross-jurisdictional data collaborations.

At a broader level, the lexicon reflects and enables current trends in genomic data sharing, including federated and privacy-preserving architectures, secure cloud-based platforms, and greater interoperability and regulatory compliance. By focusing on *what* is being done—rather than *how* or *how well*—the definitions remain relevant even as infrastructures evolve. In this sense, the lexicon serves not only to describe existing practices but also to future-proof policy discussions and technical design in an area of rapid development.

This definitional coherence also has tangible implications for data architecture and research practices in genomics. *Data visiting* has emerged as a promising approach to data sharing in genomics research. Unlike traditional data sharing, where large datasets are downloaded before being analyzed in the recipient’s environment, *data visiting* brings the analysis to the data [43]. From a data management perspective, *data visiting* helps avoid unnecessary duplication of data, solving the problem of “yet another database” (YAD) [44]. Traditionally, genomics researchers often constructed vast new datasets by copying and integrating multiple data sources for a project [45]. In the *data visiting* paradigm, analyses can be performed on multiple datasets worldwide without moving the data. This practice is especially valuable in *federated data analysis*, which allows for simultaneous analysis of datasets housed in different locations, a model well-established in other fields of science.

*Federated data analysis* is particularly useful for global collaborations as it offers control to data providers without sacrificing opportunities for collaboration and openness [46]. This method facilitates the integration of insights from multiple sources, leading to more robust and comprehensive studies. However, the complexity of coordinating between different computing environments, legal frameworks, and data standards—which is essential to perform federated data analysis—presents challenges that require sophisticated infrastructure and close collaboration [47]. 

From a security perspective, keeping data within the data provider’s computing environment can reduce the risks associated with external control of sensitive data, hence offering security benefits [48]. However, this does not automatically reduce these risks, as it also requires the provider to implement and maintain rigorous security controls—demanding significant expertise, infrastructure, and operational costs. In many cases, the most effective way to mitigate security risks is to outsource these responsibilities to a specialized third-party provider. As stated above, given that the third party is contracted by the data provider and effective control of the data remains with the data provider, such an outsourcing strategy would still be deemed the provider’s computing environment.

A controlled research setting with technical security measures like download restrictions can be established to house the shared data. These environments are often referred to as Trusted Research Environments (TREs) or Secure Research Environments (SREs), though terminology varies by region, and the terms can sometimes be used interchangeably [49]. TREs—sometimes also called data safe havens, secure data environments, or sensitive data services—are specifically designed to enable secure research while safeguarding sensitive data [50]. Typically, these environments are equipped with strong encryption, strict access controls, and comprehensive logging and monitoring systems to track user activities and prevent unauthorized access, ensuring compliance with regulations like the General Data Protection Regulation (GDPR) in the European Union (EU) or Health Insurance Portability and Accountability Act (HIPAA) in the United States (US) [51, 52]. 

In developing its lexicon, the GA4GH Data Visiting Study Group made two deliberate choices. First, although some authors wanted to include terms like “secure” or “trusted” in defining *data visiting*, these terms were intentionally excluded, acknowledging that data visiting can in practice vary widely in technical security—from minimal (if any) to robust security measures. The lexicon’s purpose is not to prescribe best practices in genomic data visiting security, but to establish a shared terminology that enables discussions about such best practices. Second, following on from this approach, it was deemed unnecessary for the data visiting lexicon to define terms that involve evaluative judgment, such as TRE or SRE.

Where fewer technical security measures are in place, contractual agreements can help ensure compliance, such as requiring visitors to refrain from unauthorized downloads. Additionally, data visiting supports the accountability principle and privacy-by-design in networked environments, as it enables the data provider to remain reasonably accountable for the data’s security and responsible use throughout its lifecycle.

*Data visiting*, if done with sufficient security safeguards, offers potential legal benefits, as it may be seen—depending on the jurisdiction—as avoiding cross-border data transfer. This could allow compliance with data localization rules without triggering stringent cross-border transfer regulations. As a result, it would help reduce the legal and transactional costs of compliance. After the analysis is completed, the provider could review the results to ensure they do not contain personal data before allowing the user to download them—further safeguarding privacy.

Together, the definitions form a structured typology that allows nuanced distinctions between real-world implementations of data access. For example, by situating *remote data interrogation* as a subset of *data visiting*, the lexicon supports clear communication about different levels of access—whether users can query data only or also view and interact with it. This internal structure reinforces consistency across terms and facilitates modular thinking about technical and governance design, allowing implementers to select the model that best matches their use case while remaining within a coherent conceptual framework. *Remote data interrogation*, in particular, is especially valuable where legal or privacy constraints prohibit direct access to sensitive data, such as patient genomic information. In such cases, the model enables international collaboration without transferring the data, provided it is implemented in a way that ensures compliance. It also allows companies to protect proprietary datasets while still enabling external research contributions [47]. 

## Conclusion

The new terms that are now included in the GA4GH lexicon mark a significant advancement in streamlining communication within the genomics community. By providing clear, standardized definitions for key concepts such as *data visiting*, *remote data interrogation*, and *federated data analysis*, the Data Visiting Study Group of GA4GH aims to foster a shared understanding across borders and institutions. These terms offer a framework to subsequently address critical legal issues such as data localization and cross-border transfer, which can impede the sharing of data. However, while consistent terminology is essential, it cannot resolve all challenges on its own. In particular, in-depth, multi-jurisdictional Ethical, Legal, and Social Implications (ELSI) research is necessary to determine whether *data visiting* constitutes *cross-border data transfer* under various legal and ethical regimes [53]. This analysis needs to embrace the entire technical security spectrum of data visiting—from minimal to high security versions of data visiting, such as *remote data interrogation*. Resolving this question will be crucial for navigating legal compliance in different contexts. Despite these unresolved issues, the promotion of standardized terminology supports clearer communication and more aligned practices in data sharing, ultimately contributing to more legally resilient data governance practices and the ongoing advancement of genomic science and health innovation.

## Electronic Supplementary Material

Below is the link to the electronic supplementary material.

**Supplementary Material 1**: **Appendix 1**

## Data Availability

No datasets were generated or analyzed during the current study.

## Abbreviations

GA4GH: Global alliance for genomics and health.
TRE: Trusted research environment.
SRE: Secure research environment.
GDPR: General data protection regulation.
HIPAA: Health insurance portability and accountability act.
YAD: Yet another database.
ELSI: Ethical, legal, and social implications.
EU: European union.
US: United States.

## References

[1] Amorim M, Silva S, Maia T, de Freitas C. Benefits and risks of sharing genetic data for research purposes: the views of patients and carers. Eur J Pub Health, 30, Issue Supplement_5, September 2020;ckaa165.365, 10.1093/eurpub/ckaa165.365

[2] Villanueva AG, Cook-Deegan R, Robinson JO, McGuire AL, Majumder MA. Genomic Data-Sharing practices. J Law Med Ethics. 2019;47(1):31–40. 10.1177/1073110519840482 30994063 10.1177/1073110519840482 PMC6730666

[3] Global Alliance for Genomics and Health (GA4GH). https://www.ga4gh.org 10.1089/gtmb.2014.1555 24896853

[4] Joly Y, Dove E, Knoppers BM, Nicol D. The GA4GH regulatory and ethics work stream (REWS) at 10: an interdisciplinary, participative approach to international policy development in genomics. In: Corrales Compagnucci M, Minssen T, Fenwick M, Aboy M, Liddell K, editors. The law and ethics of data sharing in health sciences. Singapore: Springer Nature Singapore; 2024. pp. 13–32. 10.1007/978-981-99-6540-3_2

[5] Kraemer J, Shekhar S, Hofmann J. Regulating algorithmic learning in digital platform ecosystems through data sharing and data siloing: Consequences for innovation and welfare. ICIS 2021 Proceedings (2021).

[6] Bach F, Soltau K, Göller S, Bonatto Minella C, Hofmann S. Current developments in the research data repository RADAR. Preprint at 10.3897/arphapreprints.e109429 (2023).

[7] Casaletto J, Bernier A, McDougall R, Cline MS. Annu Rev Genom Hum Genet. 2023;24:347–68. Federated Analysis for Privacy-Preserving Data Sharing: A Technical and Legal Primer. 10.1146/annurev-genom-110122-084756 PMC10846631 37253596

[8] Hallock H, et al. Federated networks for distributed analysis of health data. Front Public Health. 2021;9:712569. 34660512 10.3389/fpubh.2021.712569 PMC8514765

[9] Kairouz P, et al. Advances and open problems in federated learning. FNT Mach Learn. 2021;14:1–210.

[10] Weise M, Kovacevic F, Popper N, Rauber A. Data Sci J. 2022;21:4. OSSDIP: Open Source Secure Data Infrastructure and Processes Supporting Data Visiting.

[11] Jussen I, Schweihoff J, Dahms V, Möller F, Otto B. Data Sharing Fundamentals: Definition and Characteristics. in (2023).

[12] Government of the Australian Capital Territory. ACT Data Sharing Policy. (2025) Available from: https://www.act.gov.au/__data/assets/pdf_file/0003/2543628/ACT-Data-Sharing-Policy.pdf

[13] Mascalzoni D, Dove ES, Rubinstein Y, Dawkins HJ, Kole A, McCormack P, Woods S, Riess O, Schaefer F, Lochmüller H, Knoppers BM. International charter of principles for sharing bio-specimens and data. Eur J Hum Genet. 2015;23(6):721–8. 25248399 10.1038/ejhg.2014.197 PMC4795058

[14] Swales L, Gooden A, Thaldar D. The anatomy of a data transfer agreement for health research. Front Pharmacol. 2024;13:1332700. Available from: 10.3389/fphar.2024.1332700 10.3389/fphar.2024.1332700 PMC11383768 39257393

[15] Thaldar D, Botes M, Swales L, Esselaar P. Enhancing data governance in collaborative research: introducing SA DTA 1.1. S Afr J Bioeth Law. 2024;17(2):e2300. 10.7196/SAJBL.2024.v17i2.2300[E] 10.7196/sajbl.2024.v17i2.2300 PMC12520612 41099010

[16] National Institutes of Health (NIH). NIH Grants Policy Statement: Sect. 1.2 Definition of Terms. Bethesda (MD): NIH; 2024 Apr [cited 2025 Apr 21]. Available from: https://grants.nih.gov/grants/policy/nihgps/html5/section_1/1.2_definition_of_terms.htm

[17] UK Biobank. Enable your research. UK Biobank. Available from: https://www.ukbiobank.ac.uk/enable-your-research

[18] National Institutes of Health. All of Us Research Hub. All of Us. Available from: https://www.researchallofus.org/

[19] Australian Genomics. Genomics and privacy law. Australian Genomics. Available from: https://www.australiangenomics.org.au/genomics-and-privacy-law/

[20] GA4GH, ELIXIR, Cloud. & AAI for human data. Global Alliance for Genomics and Health. Available from: https://www.ga4gh.org/driver_project/elixir-cloud-and-aai-for-human-data/

[21] Apheris. What are collaborative data ecosystems?. Apheris. Available from: https://www.apheris.com/resources/blog/what-are-collaborative-data-ecosystems

[22] Thorogood A, et al. International federation of genomic medicine databases using GA4GH standards. Cell Genomics. 2021;1:100032. 35128509 10.1016/j.xgen.2021.100032 PMC8813094

[23] Apheris. Federated Data Networks: Enabling Cross-Institution Research. 2025 Jan 15 [cited 2025 Apr 21]. Available from: https://www.apheris.com/resources/blog/federated-data-networks

[24] National Institutes of Health (NIH). The NHGRI Genomic Data Science Analysis, Visualization, and Informatics Lab-space (AnVIL) (U24). 2017 Jul 17 [cited 2025 Apr 21]. Available from: https://grants.nih.gov/grants/guide/rfa-files/rfa-hg-17-011.html

[25] Brazil. General Data Protection Law (LGPD). 2018. Article 33. Available from: https://lgpd-brazil.info/chapter_05/article_33

[26] European Union. Regulation (EU) 2016/679 General Data Protection Regulation (GDPR). Article 44. Available from: https://gdpr-info.eu/chapter-5/

[27] India. Digital Personal Data Protection Act. 2023. Section 17. Available from: https://www.meity.gov.in/static/uploads/2024/06/2bf1f0e9f04e6fb4f8fef35e82c42aa5.pdf

[28] Israel, Privacy Protection Regulations (Data Transfer Abroad)., 2001. Regulations 1–2. Available from: https://www.gov.il/BlobFolder/legalinfo/datatransferredisrael2023/en/Privacy Protection Regulations (Instructions for Data that was transferred to Israel from the European Economic Area).pdf

[29] Kenya, Data Protection, Act. 2019. Section 48. Available from: http://kenyalaw.org/kl/fileadmin/pdfdownloads/Acts/2019/TheDataProtectionAct__No24of2019.pdf

[30] Mexico, Federal Law on Protection of Personal Data Held by Private Parties (LFPDPPP)., 2010. Articles 36–37. Available from: https://www.dlapiperdataprotection.com/?t=transfer%26c=MX#insight

[31] New Zealand. Privacy Act 2020. Principle 12. Available from: https://www.privacy.org.nz/responsibilities/disclosing-personal-information-outside-new-zealand/decision-tree-page/

[32] Nigeria, Nigeria Data Protection Act., 2023. Section 41(1). Available from: https://placng.org/i/wp-content/uploads/2023/06/Nigeria-Data-Protection-Act-2023.pdf

[33] South Africa. Protection of Personal Information Act (POPIA). 2013. Section 72. Available from: https://www.gov.za/sites/default/files/gcis_document/201409/3706726-11act4of2013protectionofpersonalinforcorrect.pdf

[34] South Korea. Personal Information Protection Act (PIPA), 2011 (as amended). Article 28. Available from: https://www.pipc.go.kr/eng/user/lgp/law/lawDetail.do#none

[35] Turkey L. No. 6698 on Protection of Personal Data, 2016 (KVKK). Article 9. Available from: https://www.fabric-ate.com/turkish-personal-data-protection-law-no-6698/

[36] United Kingdom. Data Protection Act 2018 (UK GDPR). Article 44. Available from: https://gdprhub.eu/Article_44_GDPR

[37] European Data Protection Board. Guidelines 05/2021 on the Interplay between the application of Article 3 and the provisions on international transfers as per Chapter V of the GDPR. Brussels: EDPB. 2021 Nov 19 [cited 2025 Apr 21]. Available from: https://www.edpb.europa.eu/our-work-tools/our-documents/guidelines/guidelines-052021-interplay-between-application-article-3_en

[38] Raz A, et al. Old and new challenges regarding comparable and viable data sharing in population-scale genomic research. Eur J Hum Genet. 2023;31:617–8. 36997678 10.1038/s41431-023-01355-3 PMC10250369

[39] Beck T, Hastings RK, Gollapudi S, Free RC, Brookes AJ. GWAS central: a comprehensive resource for the comparison and interrogation of genome-wide association studies. Eur J Hum Genet. 2014;22(7):949–52. 24301061 10.1038/ejhg.2013.274 PMC4060122

[40] Lancaster O, Beck T, Atlan D, Swertz M, Thangavelu D, Veal C, et al. Cafe variome: general-purpose software for making genotype–phenotype data discoverable in restricted or open access contexts. Hum Mutat. 2015;36(10):957–64. 26224250 10.1002/humu.22841

[41] Stark Z, Glazer D, Hofmann O, Rendon A, Marshall CR, Ginsburg GS, et al. A call to action to scale up research and clinical genomic data sharing. Nat Rev Genet. 2025;26(2):141–7. 39375561 10.1038/s41576-024-00776-0

[42] Baynam G, et al. Advancing diagnosis and research for rare genetic diseases in Indigenous peoples. Nat Genet. 2024;56:189–93. 38332370 10.1038/s41588-023-01642-1 PMC11229440

[43] Chen Y, Song L. China: concurring regulation of cross-border genomic data sharing for statist control and individual protection. Hum Genet. 2018;137:605–15. 30014187 10.1007/s00439-018-1903-2 PMC6132628

[44] Stark Z, et al. A call to action to scale up research and clinical genomic data sharing. Nat Rev Genet. 2024. 10.1038/s41576-024-00776-0 39375561 10.1038/s41576-024-00776-0

[45] Den Dunnen JT. Yet another database? Hum Mutat. 2018;39:755–755. 29645308 10.1002/humu.23429

[46] Choudhury S, Fishman JR, McGowan ML, Juengst ET. Big data, open science and the brain: lessons learned from genomics. Front Hum Neurosci 2015;8. 10.3389/fnhum.2014.00239 PMC4032989 24904347

[47] Atalaia A, et al. EURO-NMD registry: federated FAIR infrastructure, innovative technologies and concepts of a patient-centred registry for rare neuromuscular disorders. Orphanet J Rare Dis. 2024;19:66. 38355534 10.1186/s13023-024-03059-3 PMC10865673

[48] Sheldon J, Marietta GA et al. USA, 2024)Genomics Cybersecurity Concerns, Challenges, and a Modular Test Lab. in Proceedings of the. 10.1145/3603287.3651215

[49] Holloway L, et al. People RDC National pathfinder project: exploring federated learning tools, opportunities and resource requirements. Report to ARDC. Australian Res Data Commons. 2024. 10.5281/zenodo.13831453

[50] Weise M, Rauber A. Int J Digit Curat. 2024;18. 10.2218/ijdc.v18i1.939. Trusted Research Environments: Analysis of Characteristics and Data Availability.

[51] Goddard M, The, EU General Data Protection Regulation (GDPR). European regulation that has a global impact. Int J Mark Res. 2017;59:703–5.

[52] Chen JQ, Benusa A. HIPAA security compliance challenges: the case for small healthcare providers. Int J Healthc Manag. 2017;10:135–46.

[53] Hallinan D, et al. International transfers of personal data for health research following Schrems II: a problem in need of a solution. Eur J Hum Genet. 2021;29:1502–9. 33953344 10.1038/s41431-021-00893-y PMC8099706

