# Supplementary material for: Communicating clearly about data sharing in genomics
Source: Hum Genomics. 2025 Jul 14;19:80. doi: 10.1186/s40246-025-00784-z (PMC12257658; doi:10.1186/s40246-025-00784-z)
Supplement: Supplementary file 1 — Supplementary Material 1: Appendix 1 [file 40246_2025_784_MOESM1_ESM.docx]

**Appendix 1**

**Data Visiting Lexicon**

**Data sharing** is where one party, the provider, provides access to data to another party, the user, with the purpose of the user using the data, as agreed with the provider.

.

**Shared data** is the data that is the subject of data sharing.

**Data visiting** is a form of data sharing in which an analysis is performed on **shared data** within the provider’s computing environment, whether through human or computational agents.

**Federated data analysis** refers to **data visiting** involving multiple providers.

**Cross-border data transfer** is the transfer of data across jurisdictions.

**Remote data interrogation** is a restricted type of data visiting where the user can only query but not view the **shared data** within the computing environment of the provider and can only view the results of the query.

**Data localization** refers to legal or ethical rules, or efforts to comply with such rules that aim to keep data within a jurisdiction that governs such data.
